# SerumTrace Elements in Febrile Seizure: A Case-Control Study

**Published:** 2016

**Authors:** Kokab NAMAKIN, Mahmoud ZARDAST, Golamreza SHARIFZADEH, Toktam BIDAR, Samaneh ZARGARIAN

**Affiliations:** 1 Birjand atherosclerosis and coronary artery research center, Birjand University of Medical Sciences (BUMS), Birjand, Iran; 2 Birjand atherosclerosis and coronary artery research center, Department of pathology, Birjand University of Medical Sciences (BUMS), Birjand, Iran.; 3 Department of Social Medicine, Birjand University of Medical Sciences, Birjand, Iran.; 4 Researcher, Birjand University of Medical Sciences, Birjand, Iran

**Keywords:** Zinc, Sodium, Calcium, Magnesium, Febrile convulsion, Children

## Abstract

**Objective:**

Febrile seizure (FS) is one of the most common neurological problems during childhood.Pathogenesis of febrile convulsion is unknown. This study investigated some trace elements among children admitted with FS compared with thoseof febrile without seizure attacks.

**Materials & Methods:**

This case-control study was conducted on48 children (6 months to 5 yrold) diagnosed with febrile seizure as the cases and 48 age-matched febrile children as the control group. Serum levels of magnesium, calcium, sodium, potassium, and serum zinc were measured. Statistical analysis was performed with SPSS (version 15) using Student t-test.

**Results:**

There were no significant differences between the cases and controls in terms of gender or age. The means of serum level of zinc, sodium, calcium and magnesium in the case group was lower than those of the control group. There was no significant difference onserum potassium mean level between the case and control groups.

**Conclusion:**

Deficiency of trace elements was correlated significantly with febrile convulsion, while further investigations on trace elements are required.

## Introduction

Febrile seizure (FS) is a highly common neurological problem at childhood (1). Approximately, 2%-5% of children are estimated to undergo at least one seizure during a febrile illness before they get 5 yrold (2), accounting for 30% of all seizures among children. Seizure is associated with feveralthough there is no evidence of intracranial infection or a definite cause for it (1, 2). The mechanisms underlying FS have multifactorial etiology, complicated by the fact that the pathogenesis of FS is unknown in most cases. FS represents the point between a low seizure threshold and genetic components. Several essential elements play important roles in redox reactions, in connective tissue or cell membranes, in stabilization of biological molecules, and in control of biological processes by facilitating the binding of molecules to receptor sites on cell membranes (3). While disturbance in serum electrolytes is considered as a pathogenetic theory of FS, it has not been confirmed as yet. Low levels of someelements such as iron and sodium (Na) in the blood play roles in repeated occurrence of FS (4). We aimed to investigate some trace elements among children admitted with FS compared with thoseof febrile without seizure attacks.

Materials & Methods This case-control study, conducted in a hospital in eastern Iran, enrolled96 individuals including 48 children aged6 months to 5 yrdiagnosed with febrile convulsion (FC) as the case group, and 48 age-matched children with fever but without seizure as the controls who were from the same setting. Inclusion criteria for the cases were patient with fever (≥ 38 ºC) and a history of seizure within the last 6 h, normal cerebrosspinal fluid examination,no metabolic disorder, no intake of serum, and no pneumonia, gastroenteritis, kidney, cardiac, or developmental disorder. The control group had the same criteria except for seizure. On the other hand, the patients who had no evidence of central nervous system infection, epilepsy, metabolic seizures, or those who were already on zinc therapy or other elements for any other ailment such as malnutrition, diarrhea, pneumonia or acrodermatitisenteropathica were excluded. Blood samples (5 ml) were taken on the first hours of admission for zinc (Zn), magnesium (Mg), calcium (Ca), sodium (Na) and potassium (K). Serum levels of Mg, Ca, Na, and K were measured usinga photometric method using an auto analyzer device, and serum Zn was measured by atomic absorption spectrophotometry. The exact goal of the project was explained to the parents of the children and informed consents were taken from them. Local Ethics Committee confirmed the process. Statistical analysis was performed usingSPSS version 15 (Chicago, IL, USA) using Student t-test. Data were expressed as the mean ± standard deviation (SD). The significant level was set for P<0.05.

## Results

There were no significant differences between the cases and controls with regard to gender (P=0.64) or temperature (P=0.084) (Table 1). The mean age of FS cases was 24.1±13.4 months and that of the controlgroup without FS was 19.8±11.1 months, indicating a statistically insignificant difference (P=0.09). Besides, serum mean levels of zinc, Na, Ca and Mg in the case group were lower than those of the control group (Table 2). There was no significant difference in the serum mean K level between the case and control groups (4.38±0.41 and 4.5±0.51 respectively) (Table 2).

**Table 1:** Comparison between Cases and Controls regarding Age and Gender

** Group **	**Cases (n=48)**	**Controls (n=48)**	***P*** **-value**
**variable**			
**Age (months)**	24.1±13.4	19.8±11.1	*P*=0.09
**Sex**			
**Male** **Female**	18 (37.5%) 30 (62.5%)	20 (41.7%) 28 (58.3%)	*P*=0.68

**Table 2:** Comparison between Cases and Controls regarding Laboratory Data

** Group**	**Cases (n=48)**	**Controls (n=48)**	**Independent ** ***t*** **-test**
**Variable**			
**Na**	136.2±3.3	140±3.9	*P*<0.001
**K**	4.38±0.41	4.5±0.51	*P*<0.22
**Ca**	8.79±0.47	9.17±0.59	*P*<0.001
**Zn**	80.5±21.7	117.2±35.5	*P*<0.001
**Mg**	1.9±0.32	2.27±0.38	*P*<0.001

## Discussion

FS is the most common cause of seizures among children. It has been known since ancient times that seizures frequently accompany fever in young children. The exact pathogenesis is unknown but involves factors such as genetic predisposition and alterations. In the present study, we investigated the levels of trace and major element concentrations among children with FS. Ourresults showed that bio-element levels were affected in children with FS. The changes in bioelements in FS explained the response of the metabolism. Zinc is a component of more than 300 different enzymes that functions in many aspects of cellular metabolism, involving metabolism of proteins, lipids, and carbohydrates (5). It is believed that Zn as a co-factor of glutamic acid decarboxylase modulates the production of gammaaminobutyric acid in the central nervous system. It modulates the activity of glutamic acid decarboxylase, which is a rate limiting enzyme in the synthesis of gammaaminobutyric acid (GABA). Furthermore, it increases the affinity of neurotransmitters such as glutamate to their receptors and facilitates the inhibitory effect of Ca on N-methyld-aspartate receptors (6). Mg is involved in neuronal functions and inhibits the facilitatory effects of Ca on synaptic transmission. It exerts a voltage dependent blockage of Nmethyl-Daspartate (NMDA) receptor channel (6). In this study, a significantly low serum Zn and Mg level was found in patients with febrile convulsion as compared with the controls. Similar findings have been reported earlier, which found that the mean serum concentration of Mg and Zn were significantly lower in the children with febrile convulsion (2, 3, 7, 8-10). In this line, the serum Zn level was found significantly lower in cases of simple febrile seizures than in controls (11, 12), while Sadeghzadeh et al. did not found any clear abnormalityin serum, Ca or Zn levels in children with FS although his study did not have a control group (13). In a casecontrol study, serum Zn level of cases was lower than that of controls; however, the reduction in its level was not statistically significant (14). Hypozincemia triggers the NMDA receptor which is one of the members of glutamate family receptors. It can stand to reason, therefore, hypozincemia may play an important role in initiation of epileptic discharge (14). In the current study, a significantly low Ca concentration was found in patients with febrile convulsion as compared with the controls and K concentrations were different in the two groups. Ca and K concentrations in the FS group were lower than in the control group (3). We found a significant difference between the mean serum sodium of children with FS and controls. This is contrary to Nadkarni et al. results (6) but in line with Heydarian et al.’s (15). While this was a case–control study with a sound design, it suffered from some shortcomings such as we did not perform the detection of CSF level of the trace elements.

In conclusion, serum Zn, Na, Ca, and Mg levels were significantly lower in children with simple febrileseizure in comparison with febrile children without seizure. It can emphasize the hypothesis that there is a relation between some serum elements’ levels and febrile seizure in children.
